# Numerical Investigation of the Particle Dynamics in a Rotorgranulator Depending on the Properties of the Coating Liquid

**DOI:** 10.3390/pharmaceutics15020469

**Published:** 2023-01-31

**Authors:** Philipp Grohn, Stefan Heinrich, Sergiy Antonyuk

**Affiliations:** 1 Institute of Particle Process Engineering, University of Kaiserslautern-Landau, Gottlieb-Daimler-Straße 44, 67663 Kaiserslautern, Germany; 2 Institute of Solids Process Engineering and Particle Technology, Hamburg University of Technology, Denickestraße 15, 21073 Hamburg, Germany

**Keywords:** CFD-DEM simulation, wet particles, capillary force, viscous force, fluidized bed rotor granulator

## Abstract

In the pharmaceutical industry, the coating of particles is a widely used technique to obtain desired surface modifications of the final product, e.g., controlled release of the active agents. The production of round, coated particles is particularly important, which is why fluidized bed rotor granulators (FBRG) are often used for this process. In this work, Computational Fluid Dynamics (CFD) coupled with the Discrete Element Method (DEM) is used to investigate the wet particle dynamics, depending on the properties of the coating liquid in a FBRG. The DEM contact model was extended by liquid bridge model to account for capillary and viscous forces during wet contact of particles. The influence of the relative contact velocity on the maximum length of the liquid bridge is also considered in the model. Five different cases were compared, in which the particles were initially wetted, and the liquid loading as well as the surface tension and viscosity of the liquid were changed. The results show that increasing viscosity leads to a denser particle bed and a significant decrease in particle rotational velocities and particle motion in the poloidal plane of the FBRG. Reducing the liquid loading and surface tension results in increased particle movement.

## 1. Introduction

For various products in the pharmaceutical, chemical and food industries, the coating of particles is an important processing step in order to obtain desired surface modification of the final product [1,2]. Numerous coating equipment exists for this purpose. The coating devices can be distinguished according to their method of introducing kinetic energy into a particle bed, between a purely mechanical input (e.g., mixer, disc and drum granulators) and a fluidization induced by the energy of the process gas flow (e.g., fluidized bed or spouted bed systems). Particularly in the pharmaceutical industry, fluidized bed rotor granulators (FBRG) are widely used to produce round coated pellets for oral drug delivery with a narrow size distribution, high strength, smooth surface and high sphericity [3,4,5,6,7]. This is achieved by the special design of a FBRG. It consists of a rotating circular rotating base plate and a stationary cylindrical wall. The fluidization gas flow passes through an annular gap between the rotating plate and the cylindrical wall. This combination enables the individual process steps of spheronization, coating and drying to be carried out in the same unit [8].

Although the technology of a FBRG is widely used, the particle dynamics are still not fully understood due to the complex micro mechanisms in the process. Several experimental studies can be found in the literature that describe some mechanisms during the granulation process [4,6,9,10,11]. However, the knowledge in this field is mainly empirical and all the particle interactions are not yet fully understood. A detailed knowledge of the particle motion on the micro level is required to better understand the coating process in the rotor granulator. Numerical simulations are particularly suitable for this purpose [5,8]. The widely used Euler–Lagrange approach can be applied to simulate the multiphase flow, where Computational Fluid Dynamics (CFD) is coupled with the Discrete Element Method (DEM) [12,13]. In CFD, the flow field of the gas in the process is calculated treating the fluid phase as a continuum. For the DEM, the interactions of each particle are determined based on contact models describing the physical properties of the particles, such as adhesion, and their mechanical behavior under slow, fast and repeated loading. In two-way CFD-DEM coupling, both the influence of the gas phase on the particle phase and the influence of the particle phase on the gas phase are considered [13,14,15,16,17], while in one-way coupling, only the influence of the gas phase on the particles is taken into account [5].

The particle dynamics in a rotor granulator were first investigated by Muguruma et al. [18] numerically with DEM and experimentally with Particle Tracking Velocimetry (PTV). They studied the influence of liquid on particle motion, but considered only capillary and not viscous contact forces, and did not vary the properties of the liquid. Weis et al. [19,20] used DEM simulations to obtain the particle dynamics and mixing behavior, as well as the contact frequency of the particles in a spheronizer that, in contrast to a rotor granulator, works without fluidization air and usually at higher rotation velocities of the structured friction plate. In addition, they extended the DEM approach to consider particle rounding during this process. Recently, Grohn et al. [21] investigated numerically the multiphase flow of cylindrical particles in a FBRG with CFD-DEM simulations. A significant influence of the particle shape on the particle dynamics was found. Neuwirth et al. [4,22] performed an experimental study of the particle dynamics in a FBRG under dry and wet conditions using magnetic particle tracking (MPT). The comparison with the CFD-DEM simulations showed good agreement with the experiments for the dry case. However, the experiments were performed with particles of 6-mm diameter, which are not representative for real applications in FBRG. This particle size was required by the MPT measurement equipment available at that time. In our last study [8], the dynamics of initially wetted particles in the FBRG was investigated numerically by CFD-DEM simulations and experimentally by an improved MPT measurement system. In the numerical simulations, the capillary forces due to the presence of liquid on particles were considered based on the model of Israelachvili [23] and the viscous forces were calculated according to the models of Lian et al. [24] and Popov [25]. In addition, a new model was implemented to describe the velocity-dependent rupture length of liquid bridges [8,26]. With the improved MPT equipment, the particle dynamics of spherical particles with a minimum diameter of 2.8 mm could be measured. It was possible to validate contact models used in simulations of dry particles and particles wetted with water, and a good agreement was found.

Since in real applications, both the liquid spray rate and thus, the liquid loading of the particles and the properties of the coating solution vary, the influence of these parameters on particle dynamics and contact behavior in the FBRG are investigated in this work using the model previously validated in [8]. On the one hand, the influence of the liquid loading of 1 vol.-% and 5 vol.-% with water is investigated. On the other hand, the liquid properties are varied three times at a constant liquid loading of 5 vol.-%. The basis of this liquid is a coating solution frequently used in the pharmaceutical industry, consisting of distilled water with 6 mass-% PHARMACOAT^®^ 606 (hydroxypropyl methylcellulose, Shin-Etsu Chemical Co., Ltd., Chiyoda-ku, Tokyo, Japan) [5]. This coating solution is characterized by a reduced surface tension of 42.5 mN∙m^−1^ compared to water and a strongly increased viscosity of 61.9 mPa∙s. The three other variants thus result from: a reduction of the surface tension to the value of the coating solution while the viscosity of water remains unchanged, the surface tension of water remains unchanged but the viscosity is increased to the value of the coating solution, and both the surface tension and the viscosity are changed to the values of the coating solution. To analyze the influence of the studied liquid parameters on the particle dynamics, the distributions of solid volume fraction, tangential, poloidal and rotating particle velocities are compared. For a deeper understanding of the process, the particle contact phenomena, such as the resulting aggregate size, are investigated with DEM.

## 2. Model Description

### 2.1. CFD Modeling

In the CFD, the gas flow field is calculated by solving the volume-averaged Navier–Stokes equations [27]. For this purpose, the flow domain for the CFD simulation must first be discretized by mesh cells. In order to take the influence of the particulate phase on the gas flow into account, the volume fraction of the gas phase $\varepsilon_{g}$ in each mesh cell is included in the volume-averaged Navier–Stokes equation. Therefore, the governing equations of the mass and momentum conversation can be given as follows:

(1) $$\frac{\partial \left(\varepsilon_{g}\rho_{g}\right)}{\partial t}+\nabla \cdot \left(\varepsilon_{g}\rho_{g}{\vec{u}}_{g}\right)=0\;,$$

(2) $$\frac{\partial \left(\varepsilon_{g}\rho_{g}{\vec{u}}_{g}\right)}{\partial t}+\nabla \cdot \left(\varepsilon_{g}\rho_{g}{\vec{u}}_{g}{\vec{u}}_{g}\right)=-\varepsilon_{g}\nabla p+\nabla \cdot \left(\varepsilon_{g}{\vec{\tau }}_{g}\right)-{\vec{S}}_{p}+\varepsilon_{g}\rho_{g}\vec{g}\;,$$

where, p represents the pressure, $\rho_{g}$ describes the density, ${\vec{u}}_{g}$ and ${\vec{\tau }}_{g}$ are the velocity and the stress tensor of the gas phase, respectively. For the calculation of the volume fraction of the gas phase $\varepsilon_{g}$ in each CFD mesh cell, the volume fraction $x_{i}$ of each particle volume $V_{p,i}$ within the cell were determined using the so-called sample points approach. The principle of this method, where the volume of all particles z in a grid cell with the volume $V_{\mathrm{cell}}$ is approximated by cubic sample volumes, was first presented by Hoomans et al. [28]. In the used framework of CFDEM^®^ coupling [29], the particle is divided into 29 non-overlapping regions of equal volume, each with one sample point [30]. At each time step, the algorithm checks which of the sample cubes are located in which mesh cell:

(3) $$\varepsilon_{g}=1-\left(\overset{z}{\underset{i=0}{\sum }}x_{i}V_{p,i}\right)\frac{1}{V_{\mathrm{cell}}}\;.$$

To consider the interactions between the particulate phase and the gas phase, the momentum balance is extended by the momentum sink term ${\vec{S}}_{p}$. The momentum sink term can be determined from the drag forces ${\vec{F}}_{d,i}$ of all particles $n_{p}$ in the mesh cell with the volume $V_{\mathrm{cell}}$:

(4) $${\vec{S}}_{p}=\frac{1}{V_{\mathrm{cell}}}\cdot \overset{n_{p}}{\underset{i=0}{\sum }}{\vec{F}}_{d,i}\;.$$

Various gas–solid models can be found in the literature that describe the drag forces acting on the particles in a fluidized bed [12,31]. As in our previous work [8], the drag forces are calculated according to the widely used model of Di Felice [32], which describes the entire porosity range and for particle Reynold numbers $Re_{p,i}$ up to 10^6^ with a continuous function. Here, the drag force counteracts the relative velocity of a particle in a fluid $\left({\vec{u}}_{g}-{\vec{u}}_{p}\right)$:

(5) F→d,i=18CD,iRepρgπdp,i2u→g−u→pu→g−u→pεg2−β .

The drag force is considerably influenced by the drag coefficient $C_{D,i}$. This coefficient is related to the Reynolds number of the particles, which takes into account the superficial velocity differences between particles and the surrounding fluid [33,34]:

(6) $$C_{D,i}={\left(0.63+\frac{4.8}{\sqrt{Re_{p,i}}}\right)}^{2}\;,$$

(7) $$Re_{p,i}=\frac{\varepsilon_{g}\rho_{g}d_{p,i}\left|{\vec{u}}_{g}-{\vec{u}}_{p}\right|}{\eta_{f}},$$

where, $\eta_{f}$ is the dynamic viscosity of the fluid. The influence of the particle concentration in a mesh cell on the drag force in Equation (5) is modeled with a function [32]:

(8) β=3.7−0.65exp−1.5−log10Rep,i22 .

### 2.2. DEM Modeling

In order to simulate the motion of particles, the equations of motion for translation and rotation according to Newton and Euler are solved. For this purpose, the Discrete Element Method (DEM), firstly described by Cundall and Strack [35], is applied, which also allows for an investigation of the mechanical interactions between particles and between particles and walls. For the consideration of the influence of the gas phase on the particles in the simulations, the multiphase flows have to be calculated with the two-way CFD-DEM coupling. Therefore, in this coupling method, the model of Cundall and Strack [35] is extended to incorporate the drag force, pressure gradient force ${\vec{F}}_{\nabla p,i}$ and viscous force in gas ${\vec{F}}_{\vec{\tau },i}$ [12,33,36]:

(9) $$m_{p,i}\frac{d{\vec{v}}_{p,i}}{dt}={\vec{F}}_{d,i}+{\vec{F}}_{\nabla p,i}+{\vec{F}}_{\vec{\tau },i}+{\vec{F}}_{g,i}+\sum_{j=0}^{k}{\vec{F}}_{c,\mathrm{ij}}+{\vec{F}}_{\mathrm{vis},\mathrm{ij}}+{\vec{F}}_{\mathrm{cap},\mathrm{ij}}\;,$$

(10) $$J_{p,i}\frac{d{\vec{\omega }}_{p,i}}{dt}=\sum_{j=0}^{k}\left({\vec{M}}_{t,\mathrm{ij}}+{\vec{M}}_{r,\mathrm{ij}}\right)\;.$$

The gravitational force ${\vec{F}}_{g,i}$ and the sum of the contact forces ${\vec{F}}_{c,\mathrm{ij}}$, which act on the particle due to interactions with other particles j or the walls, are modeled to determine the translational velocity of each particle ${\vec{v}}_{p,i}$ with the mass $m_{p,i}$. Similar to our latest work [8], the particles are initially wetted. Therefore, viscous forces $F_{\mathrm{vis},\mathrm{ij}}$ and capillary forces $F_{\mathrm{cap},\mathrm{ij}}$ act during a particle contact. Both forces are described in detail in the following Section 2.2.2. The sum of the torques ${\vec{M}}_{t,\mathrm{ij}}$ caused by the tangential forces acting on the particle and the torques ${\vec{M}}_{r,\mathrm{ij}}$ due to rolling friction if the particle rotates are calculated to determine the angular velocity ${\vec{\omega }}_{p,i}$ of each particle with the moment of inertia $J_{p,i}$.

#### 2.2.1. Contact Forces

The contact forces are calculated according to the well-known Hertz–Mindlin model, which is described in detail in the literature [37,38,39]. The contact force is decomposed into a normal and tangential component, index n and t, respectively, and are expressed as:

(11) $$F_{c,n,\mathrm{ij}}=-k_{n}\delta_{n}^{\frac{3}{2}}n_{\mathrm{ij}}-\eta_{n}u_{p,n,\mathrm{ij}}\;,$$

(12) $$F_{c,\mathrm{ij},t}=\left\{\begin{array}{cc} -k_{t}\delta_{t}t_{\mathrm{ij}}-\eta_{t}u_{p,t,\mathrm{ij}} & \mathrm{if}\;\left|-k_{t}\delta_{t}t_{\mathrm{ij}}-\eta_{t}u_{p,t,\mathrm{ij}}\right|\leq \mu \left|F_{c,n,\mathrm{ij}}\right|\\ -\mu \left|F_{c,n,\mathrm{ij}}\right|t_{\mathrm{ij}} & \mathrm{if}\;\left|-k_{t}\delta_{t}t_{\mathrm{ij}}-\eta_{t}u_{p,t,\mathrm{ij}}\right|>\mu \left|F_{c,n,\mathrm{ij}}\right| \end{array}\;,\right.$$

where k describes the spring stiffness coefficient, δ represents the displacement,$\;n_{\mathrm{ij}}$ as well as $t_{\mathrm{ij}}$ are normal and tangential unit vectors, $u_{p,n,\mathrm{ij}}$ and $u_{p,t,\mathrm{ij}}$ are the normal and tangential component of the contact velocity vector of the particle with another particle or a wall. The sliding friction coefficient is represented by μ. The energy dissipation due to viscoelastic deformation behavior of the material is taken into account by the damping factor $\eta_{n,t}$ for the normal and tangential direction:

(13) $$\eta_{n}=2\alpha \sqrt{m^{*}k_{n}}\delta_{n}^{1/4},$$

(14) $$\eta_{t}=2\alpha \sqrt{m^{*}k_{t}}\delta_{t}^{1/4}\;,$$

where, α represents a function of the restitution coefficient, and $m^{*}$ is the reduced mass of the contact partners. A more detailed description can be found in Heinrich et al. [17] and Salikov et al. [37].

#### 2.2.2. Capillary and Viscous Forces

**Figure 1:**
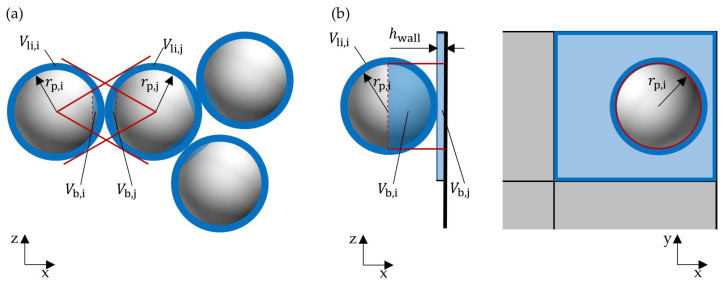
Determination of (**a**) the volume of liquid used to form the liquid bridge according to the model of Shi and McCarthy [41] and (**b**) the volume of liquid passing from a wetted grid cell of the wall to the liquid bridge.

During a wet particle contact, additional adhesive forces need to be considered. Capillary forces act due to the surface tension of the liquid, and viscous forces act due to the relative motion of particles and liquid in the liquid bridge. A wet contact begins as soon as the liquid layers on the contact partners touch and ends as soon as the maximum liquid bridge length is reached. During this contact time, the capillary and viscous forces must be taken into account [8,26]. In the literature, several models describe the capillary forces for symmetric pendular bridges [18,23,38,39,40] on the basis of the total liquid bridge energy and the pressure difference across the liquid bridge. The numerical simulation method validated in our last study [8] is also used in this work. Therefore, the capillary forces are calculated according to Israelachvili [23]. Thus, the capillary force acting between two particles $F_{\mathrm{cap},\mathrm{pp}}$ and the capillary force acting between a particle and a wall $F_{\mathrm{cap},\mathrm{pw}}$ are given by the following expressions:

(15) $$F_{\mathrm{cap},\mathrm{pp}}=\frac{-4\pi R^{*}\gamma cos\left(\theta \right)}{1+{\left(\sqrt{1+\frac{V_{b}}{\pi R^{*}h^{2}}}-1\right)}^{-1}}\;,$$

(16) $$F_{\mathrm{cap},\mathrm{pw}}=\frac{-8\pi R^{*}\gamma cos\left(\theta \right)}{1+{\left(\sqrt{1+\frac{V_{b}}{\pi R^{*}h^{2}}}-1\right)}^{-1}}\;,$$

where, γ represents the surface tension, θ is the wetting angle, $V_{b}$ describes the volume of the liquid bridge and h is the shortest distance between the particles or the particle and the wall. $R^{*}$ represents the effective contact radius, which is expressed as:

(17) $$R^{*}=\frac{r_{i}r_{j}}{r_{i}+r_{j}}\;,$$

where, $r_{i}$ and $r_{j}$ are the radii of the two contact partners. The assumption is made that the liquid on the particles forms a uniform thin film over the particle surface. During a wet particle contact, a liquid bridge is formed between the contact partners in the rebound phase. Shi and McCarthy’s [41] distribution model is used to determine the liquid volume of these liquid bridges for particle–particle contacts. The distribution model ensures that the liquid on the particle surface contributes to only one liquid bridge (Figure 1a). However, this approach is only valid for monodisperse systems. The liquid volume $V_{b,i}$ that particle i contributes to the liquid bridge is then calculated as follows:

(18) $$V_{b,i}=\frac{L_{i}}{2}\cdot \left(1-\sqrt{1-\frac{r_{j}^{2}}{{\left(r_{i}+r_{j}\right)}^{2}}}\right)\;,$$

where, $L_{i}$ is the total liquid volume present on particle i. The contributed liquid volume from particle j is determined in a similar manner:

(19) $$V_{b,j}=\frac{L_{j}}{2}\cdot \left(1-\sqrt{1-\frac{r_{i}^{2}}{{\left(r_{i}+r_{j}\right)}^{2}}}\right)\;.$$

Figure 1b shows the contact case between a particle and a wetted wall. The volume of liquid contributed by the wall depends on the virtual liquid layer thickness $h_{\mathrm{wall}}$ on the surface grid cell of the wall geometry in contact. The layer thickness is calculated by the liquid volume associated with the wetted wall grid cell divided by its surface area. Often, the 2D surface grid cells of the geometry are of different sizes and often much larger than the particle surfaces. Therefore, it is assumed that only the liquid in the region corresponding to the projection area of the contacting particle needs to be considered. In Figure 1b, this area is marked with a red circle. Thus, the amount of liquid in the wall grid cell that contributes to the formation of the liquid bridge is expressed as:

(20) $$V_{b,j}=h_{\mathrm{wall}}\pi r_{i}^{2}\;.$$

The final volume of the liquid bridge is then the sum of both contributed liquid volumes:

(21) $$V_{b}=V_{b,i}+V_{b,j}\;.$$

As the particles rebound, the liquid bridge is stretched until it ruptures at a critical distance between the contact partners. This critical distance, also called the maximum liquid bridge length, is described by various models [38,39,42,43,44,45]. All models have in common that they do not consider the significant influence of the impact velocity on the bridge length, which was found in our recent experimental study [26]. In this work, three different experimental setups were developed to investigate the maximum liquid bridge length in a velocity range from 0.0001 s∙m^−1^ to 4 s∙m^−1^ for particle–particle as well as particle–wall contact. Based on our experimental results, we extended the model of Mikami et al. [39] to account for the strong influence of the impact velocity $u_{\mathrm{im},\mathrm{ij}}$ on the maximum liquid bridge length. For particle–wall contact, the maximum bridge length was expressed as:

(22) $$l_{\max,\mathrm{pw}}=\left(0.95+0.22\theta \right)V_{b}^{0.32}{\left(1+C_{\mathrm{pw}}u_{\mathrm{im},\mathrm{ij}}\right)}^{\frac{2}{3}}\;,$$

where, $C_{\mathrm{pw}}$ represents a constant parameter with was found in [26] to have a value of 4.424 s∙m^−1^. For particle–particle contact, the maximum liquid bridge length was calculated as:

(23) $$l_{\max,\mathrm{pp}}=\left(0.99+0.62\theta \right)V_{b}^{0.34}{\left(1+C_{\mathrm{pp}}u_{\mathrm{im},\mathrm{ij}}\right)}^{\frac{2}{3}}\;,$$

where, $C_{\mathrm{pw}}$ is a constant parameter with the value of 6.266 s∙m^−1^. The end of the contact is indicated by the rupture of the liquid bridge. The volume of the liquid bridge is then distributed evenly among the contact partners.

In addition to the capillary forces, viscous forces are also considered. They slow down the contact velocity during the approach phase as well as the velocity of the rebound phase after contact. Based on the Reynolds lubrication theory [46], Adams and Perchard [47] developed a model to describe the viscous force in the normal direction, which is often used in DEM simulations [24,41,48,49]. In the model, two particles are assumed to be in a liquid layer and move with a relative velocity in the normal direction. The viscous forces in normal direction $F_{\mathrm{vis},n}$ between the particles can then be calculated by the Reynolds lubrication equation:

(24) $$F_{\mathrm{vis},n}=\frac{6\pi \eta_{l}R^{*}{}^{2}u_{p,\mathrm{ij},n}}{h}\;,$$

where, $\eta_{l}$ is the dynamic viscosity of the liquid, $u_{p,\mathrm{ij},n}$ represents the relative velocity of the colliding particles or particle with a wall in normal direction and h is the shortest distance between the surfaces of the contact partners. Similar to DEM studies from other authors [48,50,51], a minimum distance between the contact partners is set for the calculation of the viscous forces as it is physically limited by the roughness of the respective surfaces. The viscous force in tangential direction is calculated according to the model of Popov [25]. It describes the tangential force acting on a spherical particle moving along a plate wetted with a liquid film:

(25) $$F_{\mathrm{vis},t}=2\pi \eta_{f}R^{*}u_{p,\mathrm{ij},t}\ln\left(1+\frac{R^{*}}{2h}\right)\;,$$

where, $u_{p,\mathrm{ij},t}$ describes the relative velocity of the colliding particles or particle with a wall in tangential direction.

## 3. Simulation Setup

### 3.1. Geometry of the Fluidized Bed Rotor Granulator

**Figure 2:**
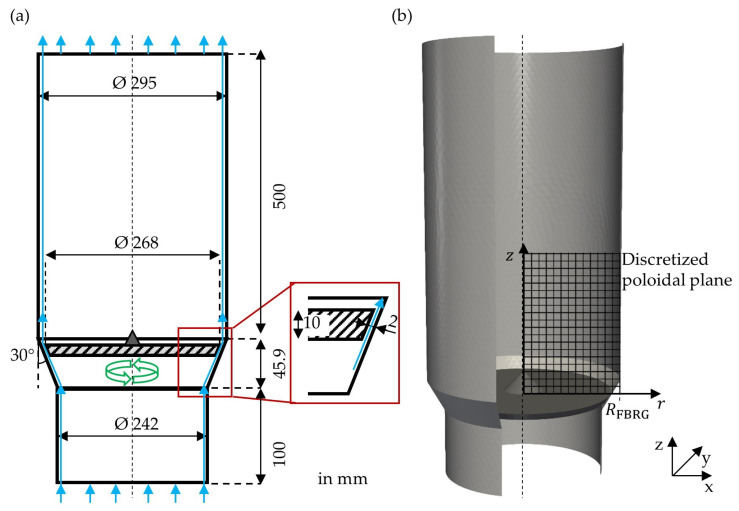
(**a**) Dimensions of the fluidized bed rotor granulator (blue arrows indicate the air flow and the green arrows the direction of rotation of the plate) and (**b**) its STL 3D geometry for the simulations.

In this study, a FBRG is investigated, whose dimensions are inspired by the commercially used rotor granulator Rotor 300 (Glatt GmbH, Binzen, Germany). In Figure 2, the geometry of the apparatus is shown. The diameter of the cylindrical process chamber is 295 mm; thus, the radius $R_{\mathrm{FBRG}}$ is 147.5 mm (Figure 2b). FBRG has an unstructured rotating plate with a diameter of 268 mm located in the middle of the apparatus. In addition, the gas flows vertically into the particle bed via a two-millimeter-wide annular gap between the rotor plate and the apparatus wall. Due to the small inflow surface of the annular gap, the gas enters the process chamber with a much higher velocity than in conventional fluidized beds. The direction of the air flow in the apparatus is shown by blue arrows and the rotation of the plate is represented by green arrows. In real applications, an additional nozzle is often placed above the plate to coat the particles, but in the simulated cases in this study, only initially wetted particles are examined.

### 3.2. Simulation Setup

For the CFD, the geometry of the FBRG was discretized into around 100,000 hexagonal mesh cells. The open-source software OpenFOAM^®^ [52] was used to solve the Navier–Stokes equations with a pressure implicit with splitting of operator algorithm (PISO method) [53]. Turbulence was included using a k-ε turbulence model [54] and the CFD time-step was $5\times 10^{-6}$ s. The fluidization gas was air at 20 °C. An operation point was simulated, where the inlet flow was 200 m^3^∙h^−1^, which corresponded to an inlet velocity of 1.21 m∙s^−1^. The air velocity in the annular gap was about 32 m∙s^−1^, which is 20 times higher than the minimal fluidization velocity of the particles. The rotor plate rotated with 100 rpm. The CFD simulation parameters are listed in Table 1.

The particulate phase was calculated with DEM using the open-source software LIGGGHTS^®^ [55] and coupled with the CFD by the open-source software CFDEM^®^ coupling [29]. Similar to previous studies, round particles with a diameter of 2.8 mm consisting of a ceramic core and a shell of polyvinyl butyral (PVB) were investigated [5,8,26,56]. Initially wetted particles with a total mass of 1 kg were generated above the rotor plate. It was assumed that the liquid on the particles was evenly distributed on the particle surface with a layer of equal thickness. Similar to our previous work [5,8], different setups were used to obtain the particle properties needed for the contact model in DEM. With a free-fall device [26,56], the restitution coefficient was determined. A Nanoindenter (Hysitron TI Premier, Bruker Corporation, Billerica, Massachusetts, USA) was used to measure the Young’s modulus, and with a Texture Analyser^®^ (TA.XTplus, Stable Micro Systems, Godalming, United Kingdom), the static as well as the rolling friction coefficients were obtained [34]. The contact angle of water was determined with a camera setup and evaluated by a MATLAB script [26]. The DEM time-step was $1\cdot 10^{-7}$ s. The initial liquid loading of the particles in the bed is varied from 1 vol.-% to 5 vol.-% distilled water. In addition, the surface tension and viscosity of the liquid are varied three times at 5 vol.-%. The basis is a coating solution frequently used in the pharmaceutical industry, consisting of distilled water with 6 mass-% PHARMACOAT^®^ 606 (hydroxypropyl methylcellulose, Shin-Etsu Chemical Co., Ltd., Chiyoda-ku, Tokyo, Japan) [8]. This coating solution is characterized by a reduced surface tension of 42.5 mN∙m^−1^ compared to water and a strongly increased viscosity of 61.9 mPa∙s. The three other cases thus result from a reduction in surface tension with no change in the viscosity of the coating liquid, no change in the surface tension of the coating liquid but an increase in viscosity, and both the change in surface tension and viscosity to the values of the coating solution. The DEM parameters can be seen in Table 2 and the performed simulation cases are listed in Table 3.

## 4. Results

The particle dynamics and contact behavior in the fluidized bed rotor granulator obtained with CFD-DEM simulations for the five cases with different liquid loading, as well as different liquid viscosity and surface tension, are compared in the following sections.

### 4.1. Particle Solid Volume Fraction

**Figure 3:**
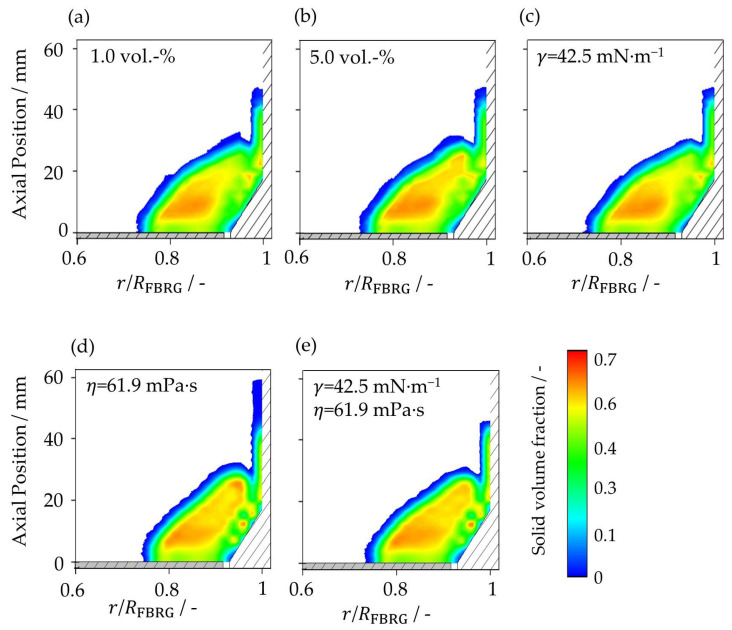
Poloidal distribution of solid volume fraction for studied cases (Table 3): (**a**) liquid loading of 1 vol.-%, (**b**) liquid loading of 5 vol.-%, (**c**) surface tension of 42.5 mN∙m^−1^, (**d**) viscosity of 61.9 mPa∙s, and (**e**) surface tension of 42.5 mN∙m^−1^ and viscosity of 61.9 mPa∙s.

First, the poloidal distribution of the solid volume fraction is analyzed (Figure 3). Figure 2b shows how the internal volume of the apparatus was discretized into 2.8 mm × 2.8 mm squares in the axial and radial directions to calculate the solid volume fraction. Every 10 ms during the steady-state periods of the simulations from 1.5 s to 2 s, the volume of particles located within this regular poloidal discretization grid was determined and averaged. In the last step, it was divided by the volume of the associated ring cell. Weis et al. [19] also evaluated the particle dynamics in a spheronizer in the same way.

The zones with high concentrations are located in the center of the particle bed, with a maximum in the area of 10 mm above the rotor plate. The increase in liquid loading from 1 vol.-% (Figure 3a) to the second case with 5 vol.-% water (Figure 3b) leads to an increase of the region with a high solid volume fraction. In the third case (Figure 3c), the decrease in surface tension also results in a reduction of the zone with high particle concentration. The poloidal distributions of the solid volume fraction in the first and third cases are very similar (Figure 3a,c). It can be seen that the two cases with a high viscosity (Figure 3d,e) differ the most from the other three cases. Here, the region with high solid volume fraction is the largest. Due to high viscosity, this region has expanded towards the apparatus wall and thus, there is also a high particle concentration in the area between 20 mm and 30 mm above the annular gap near the wall. Thereby, the particle bed has expanded the lowest in the case of the increased viscosity at constant surface tension of water (Figure 3d) and is therefore the densest. However, the particle concentration is highest at high axial positions. The reason is that the particles adhere to the wall over time and, unlike in the other cases, very rarely come off. Therefore, the concentration here increases over time. It can be clearly seen that with high liquid loading, surface tension and viscosity, and thus, high liquid bridge forces, the particle bed becomes denser.

### 4.2. Particle Velocity

**Figure 4:**
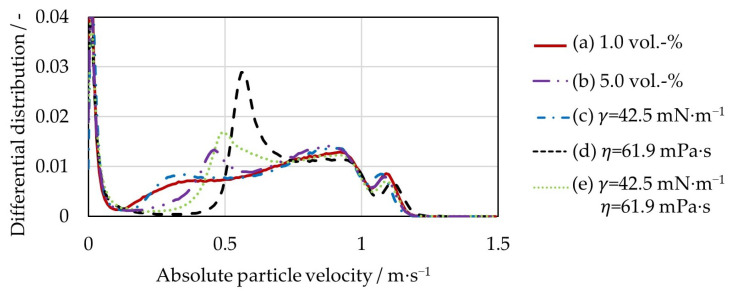
Differential distributions of the absolute particle velocity for a liquid loading of 1 vol.-%, a liquid loading of 5 vol.-%, a surface tension of 42.5 mN∙m^−1^, a viscosity of 61.9 mPa∙s, and a surface tension of 42.5 mN∙m^−1^ and a viscosity of 61.9 mPa∙s.

Figure 4 shows the differential distributions of the absolute particle velocities in the fluidized bed at different liquid loading conditions and different liquid properties. All distributions are multimodal. The first peak is at very low velocities of less than 0.01 m∙s^−1^. These are particles that are in contact with the stationary wall of the process chamber. A second peak is between 0.5 m∙s^−1^ and 0.6 m∙s^−1^. These are particles located in the upper half of the particle bed. The third peak at 1.2 m∙s^−1^ represents the particles interacting with the rotor plate. Again, a clear difference can be seen between the cases with high viscosity (curves (d) and (e)) and the other three variants with lower viscosity (curves (a)–(c)). The distributions for the first three cases are very similar (curves (a)–(c)). The liquid bridge forces decrease with lower liquid bridge volume as well as lower surface tension; therefore, less kinetic energy is dissipated during the particle contacts. Nevertheless, the average velocity of the particles with 1 vol.-% loading is 4.0% lower than that of the particles with 5 vol.-%. Also compared to the case with 5 vol.-% of water, the average particle velocity decreases by about 4.1% in the third case with a surface tension of 42.9 mN∙m^−1^. The reason is the increased slip of the particles on the rotating plate. As a result, the energy input from the rotor to the bed is lower and the mean velocity of the particles in the bed decreases slightly. This leads to a small difference between the three cases.

The increased viscosity of 61.9 mPa∙s in the fourth case (curve (d)) again leads to a lower slip, slightly increasing the mean particle velocity by 2.3%, compared to 5 vol.-% water. It can be seen that the proportion of velocities between 0.5 m∙s^−1^ and 0.6 m∙s^−1^ increases significantly. In the last case (curve (e)), the positive effect that the increased viscosity has on the slip of the particles on the rotation plate is counteracted by the negative effect of the lower surface tension. As a result, the average particle velocity only changes by less than 1%.

**Figure 5:**
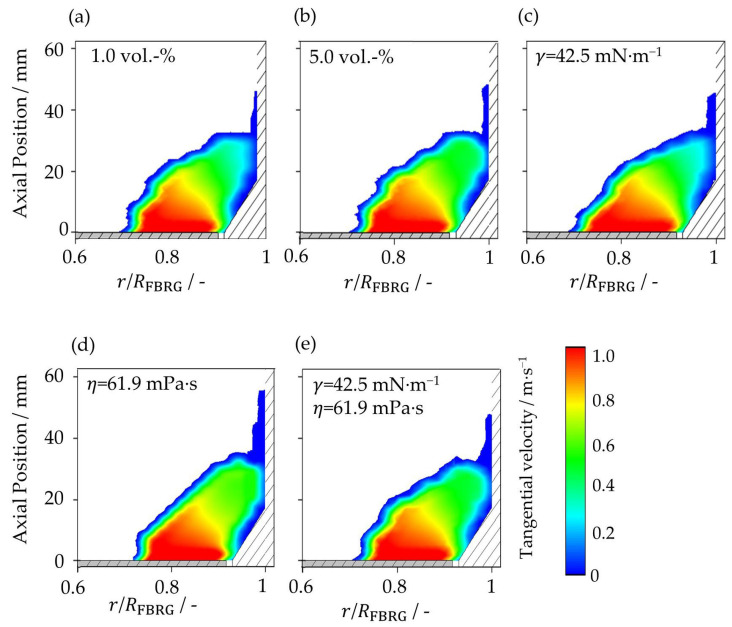
Poloidal distribution of tangential particle velocity at (**a**) liquid loading of 1 vol.-%, (**b**) liquid loading of 5 vol.-%, (**c**) surface tension of 42.5 mN∙m^−1^, (**d**) viscosity of 61.9 mPa∙s, and (**e**) surface tension of 42.5 mN∙m^−1^ and viscosity of 61.9 mPa∙s.

The tangential velocity distribution in the poloidal plane as a function of radial and axial position at a different liquid loading and at different liquid properties is shown in Figure 5. Although in all five cases, the acting liquid bridge forces differ, their velocity profiles are quite similar. The highest tangential velocities can be seen in the region directly above the rotating plate caused by transfer of momentum into the particle bed. Due to liquid bridges and, therefore, acting adhesive forces, there is a significant reduction in particle velocity near the wall in all cases investigated, as the particles repeatedly adhere to the wall. Thus, the particles are strongly decelerated and have a tangential velocity of 0 m∙s^−1^ directly at the wall. It can be seen that with an increase in the liquid bridge forces in cases (b), (d) and (e), the zone with low tangential velocities near to the stationary apparatus wall decreases. The particles are more strongly connected to each other, which improves the energy input through the rotor plate to the entire particle bed.

**Figure 6:**
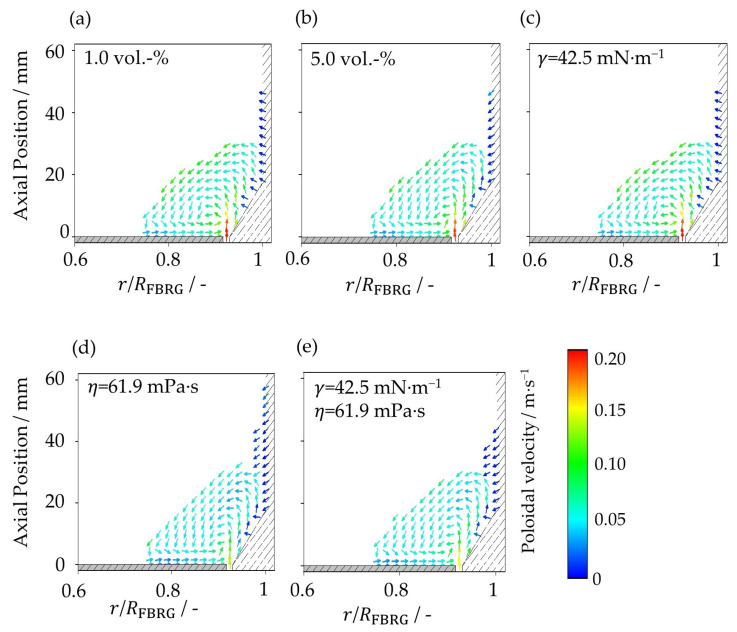
Poloidal distribution of poloidal particle velocity at (**a**) liquid loading of 1 vol.-%, (**b**) liquid loading of 5 vol.-%, (**c**) surface tension of 42.5 mN∙m^−1^, (**d**) viscosity of 61.9 mPa∙s, and (**e**) surface tension of 42.5 mN∙m^−1^ and viscosity of 61.9 mPa∙s.

**Figure 7:**
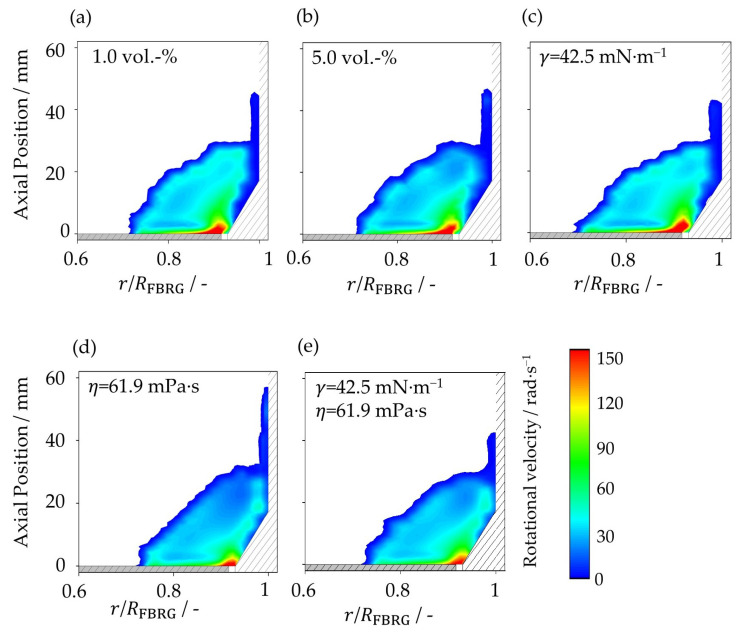
Poloidal distribution of rotational particle velocity at (**a**) liquid loading of 1 vol.-%, (**b**) liquid loading of 5 vol.-%, (**c**) surface tension of 42.5 mN∙m^−1^, (**d**) viscosity of 61.9 mPa∙s, and (**e**) surface tension of 42.5 mN∙m^−1^ and viscosity of 61.9 mPa∙s.

More pronounced differences between the five cases can be seen in the poloidal velocity distribution (Figure 6). The poloidal velocity is the velocity component composed of the z-velocity and the radial velocity [8]. It can be clearly seen that the poloidal velocities of the particles are significantly lower than their tangential velocities. The direction of particle motion in the poloidal plane is evident from the velocity vectors. Due to the rotation of the plate and the axial acceleration above the annular gap caused by the fluidization air, the particles obtain a circular movement in the poloidal plane of the particle bed. The highest poloidal velocities can be observed near the wall directly above the annular gap due to the high inflow velocity of the fluidization air. In addition, the particles have a high poloidal velocity at the surface of the particle bed, where the particles fall down by gravity. In contrast to the center of the particle bed, as well as near the apparatus wall, the particles move very slowly. The particles in the two cases with increased viscosity (Figure 6d,e) have the lowest poloidal velocities. The increased viscous forces, due to the higher viscosity of the liquid, lead to higher energy dissipation, and as a result, the poloidal velocity of the particles decreases. For the first and third case (Figure 7a,c), it can be seen that the poloidal particle velocities are slightly higher compared to the case with 5 vol.-% water (Figure 7b). Here, the lower capillary forces due to the smaller amount of liquid or the lower surface tension are responsible for the reduced energy dissipation.

For a useful description of the particle dynamics, the particle rotation number (PRN) and the radial movement proportion (RMP) can be calculated. Both parameters were developed in our previous study [8]. The particle rotation number is defined as the number of 360-degree rotations of the all particles around the central vertical axis of the process chamber per second. If, in a later case, a nozzle is installed in the wall of the process chamber to coat the particles, this key number describes how many times per second the particles pass the wet zone of the nozzle. The second number RMP describes the proportion of the radial velocity to the total velocity of the particles in the xy-plane. The higher this value, the more the kinetic energy of the particles goes into their radial motion in the bed. The values of the PRN and RMP for all simulated cases are given in Table 4. Due to the increased slip at lower liquid loading and lower surface tension (cases (a) and (c), the PRN decreases by 3.9% compared to the case (b) with 5 vol.-% of water. In contrast, it increases by 14.5% with increased viscosity (cases (d) and (e). All five cases have a significantly lower rotational speed than the rotor plate, which rotates at 1.67 s^−1^. This is mainly due to the fact that it is an unstructured plate, where the energy transfer is not as good as with structured plates [57]. Looking at the RMP, it is clear that a reduction in liquid loading leads to an increase in radial motion. This is even more pronounced for the case with reduced surface tension. In both cases, the lower capillary forces compared to 5 vol.-% water lead to a greater freedom of movement of the particles and thus to an increased poloidal velocity. As already seen in Figure 6, the velocities are lower in the poloidal plane when the viscosity is increased to 61.9 mPa∙s. The reason is due to the significant increase in viscous forces, the particles are slowed down more during contacts. As a consequence, the RMP also decreases more significantly. A lower surface tension in case e) with 6 mass-% PHARMACOAT^®^ 606 solution, and thus lower capillary forces, lead to reduced energy dissipation, which again slightly increases the RMP compared to the fourth case.

### 4.3. Rotational Particle Velocity

Another important kinematic parameter to analyze the particle dynamics is the rotational particle velocity, which describes the rotation of the particles around their center of mass. Figure 7 shows the poloidal distribution for the rotational velocity of the particles as a function of radial and axial position at different liquid loading and at different liquid properties. Directly above the rotor plate near the annular gap, the highest rotational velocities can be seen. This is due to the fact that, on the one hand, the circumferential velocity of the rotor is highest in this zone and, on the other hand, the solid volume fraction is low due to the inflowing fluidization air (Figure 3). The highest rotation velocities of the particles can be obtained in the case with 1 vol.-% water (Figure 7a). Increasing the amount of liquid as well as the viscosity increases the liquid bridge forces, which reduces the rotation velocity of the particles, whereas reduced surface tension counteracts this.

In the fourth case (Figure 7d), where the liquid bridge forces are highest, the region with high rotational velocities is smallest. The reason for this is the comparatively densest particle bed, since the mean free path length of the particles is smallest there. This in turn leads to higher adhesion rates and thus to a reduction in the rotational velocity of the particles.

### 4.4. Particle Contacts

In Table 5, the contact rates and average numbers of contact partners can be compared for the cases with different liquid loading of water as well as different liquid properties. All cases lead to a decrease in the average contact rate compared to case (b) with 5 vol.-% water. However, no significant differences can be identified between the cases. In general, a lower liquid loading as well as surface tension results in smaller liquid bridge forces. As a consequence, the particle bed is slightly less densely packed and the mean free path length, and thus the time until contact occurs again, is increased. At the same time, the average number of contact partners decreases, since the adhesive forces that lead to the formation of aggregates decrease. In the fourth case (d), with a high viscosity and unchanged surface tension, the viscous forces slow down the particle contact velocity, while capillary forces remain strongly attractive. This leads to an increase in the average number of contact partners compared to the case (b) with 5 vol.-% water, since the aggregates remain longer stable. However, this reduces the average contact rate of the individual particles. At high viscosity and lower surface tension in case (e) with 6 mass-% PHARMACOAT^®^ 606 solution, both effects counteract each other. The result is a minimally higher contact rate of the particles compared to the third case (c) and a slightly lower average number of contact partners compared to the fourth case.

**Figure 8:**
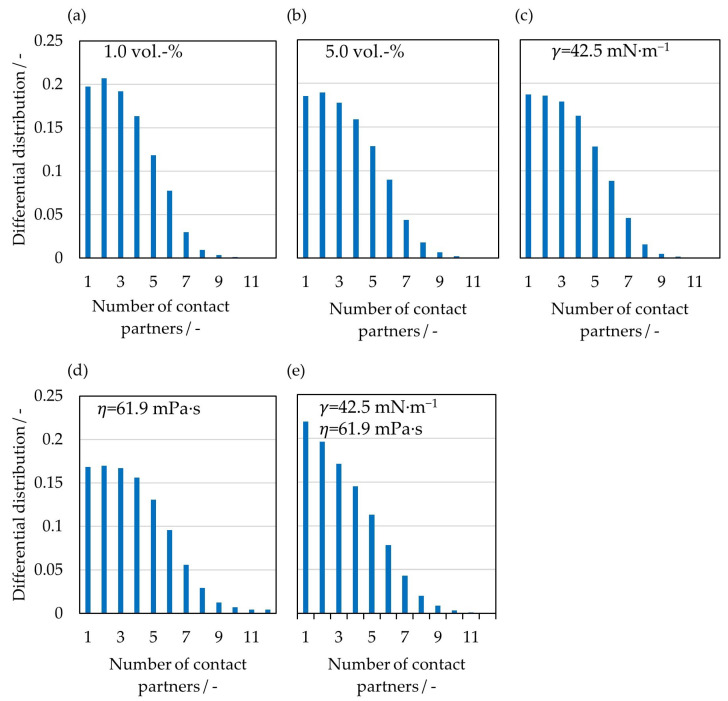
Differential distribution of the number of simultaneous contact partners for a (**a**) liquid loading of 1 vol.-%, (**b**) liquid loading of 5 vol.-%, (**c**) surface tension of 42.5 mN∙m^−1^, (**d**) viscosity of 61.9 mPa∙s, and (**e**) surface tension of 42.5 mN∙m^−1^ and viscosity of 61.9 mPa∙s.

For a more detailed analysis of the aggregates formed during the process, the differential distributions of the number of simultaneous contact partners for all five cases are shown in Figure 8. While the distributions at 1 vol.-% water (Figure 8a) and a reduced surface tension (Figure 8c) differ only slightly from the case with 5 vol.-% water, significant differences can be seen between the two cases with increased viscosity. In the fourth case (Figure 8d), the proportion of aggregates consisting of two or three particles decreases by 3.7%, while the proportion of aggregates consisting of more than seven particles increases by 4.8%. In the last case studied (Figure 8e), the proportion of aggregates consisting of two or three particles increases by 4.1%, and the proportion of aggregates of more than nine particles increases slightly as well. In all cases, the large aggregates form in the upper region of the particle bed, where the poloidal and tangential particle velocities are lowest. Since the poloidal velocities are lowest in the cases with increased viscosity, the large aggregates can exist here for the longest time before the particles separate from each other again due to shear forces.

**Figure 9:**
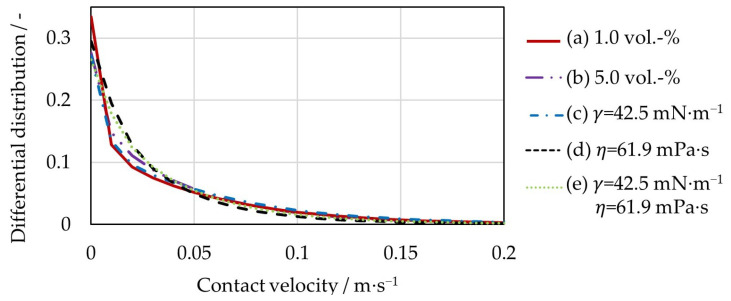
Differential distribution of the contact velocities for the five simulation cases.

In Figure 9, the differential distribution of the time-averaged contact velocities can be seen. For this purpose, the contact velocities were counted in intervals of 0.01 m∙s^−1^ and divided by the total number of contacts. Since the distribution of the contact velocities is wide, the velocity range is shown up to 0.2 m∙s^−1^ for clearer visualization. In addition, the shown range represents 98% of the occurring impact velocities. As expected, the significantly higher viscosity in the last two cases (d) and (e) leads to an increase in the proportion of low contact velocities of less than 0.05 m∙s^−1^, while the proportion of higher contact velocities decreases accordingly. The reason is the stronger viscous forces, which always oppose the motion of the particles and thus slow them down in the approach phase during contact. This can also be confirmed by the capillary number, which, in these cases, is in the range of 10^−2^, whereas in the other cases (a)–(c), it is in the range of 10^−4^. The reduction of the liquid loading (curve a)) or the surface tension (curve (c)) leads to a decrease in the attractive capillary forces. Therefore, the particles’ movement, for example PRN and RMP, in the bed is higher (Table 4). As a result, the fraction of contact velocities of more than 0.05 m∙s^−1^ is slightly greater than for case b) with 5 vol.-% water, but less than for the two cases with high viscosity.

Table 6 summarizes all average contact velocities with variation of liquid loading and liquid properties. As could already be seen from the differential distribution of the contact velocities (Figure 9), with increasing coating liquid viscosity, the fraction of low contact velocities grows. This can also be seen when looking at the average contact velocities. The highest contact velocities occur during interactions of the particles with the rotating rotor plate. In wet particle contacts, both contact forces due to viscoelastic deformation and contact forces due to liquid bridges are existent. In the FBRG, however, the contacts in which only a liquid bridge force acts predominate. Table 6 shows that the mean particle–particle contact forces, mainly contributed by liquid bridge forces, increase in both normal and tangential directions for the highly viscous cases (d) and (e). In the normal direction, the contact force increases by 14.3% for the fourth case (d) and by 4.1% for the fifth case (e) compared to the case (b) with 5 vol.-% water. When comparing the tangential force for particle–particle contacts, an even more significant increase of 76.9% in the fourth case d) and 65.4% in the fifth case e) is noticeable. The reason is that the viscous force increases proportionally with the viscosity of the liquid (Equations (24) and (25)). Since the capillary force is also proportionally dependent on the surface tension (Equations (15) and (16)), the mean contact force decreases when the surface tension is reduced. The average particle–particle contact forces in the normal and tangential directions in the case a) with reduced liquid loading and in the case (c) with lower surface tension are smaller than for 5 vol.-% water (case (b)). The ratio of the normal to the tangential contact force changes significantly for the last two cases (d) and (e) and decreases. This influence is most clearly seen for the particle–rotor contacts. Thus, the high viscosity with simultaneously reduced surface tension of the 6 mass-% PHARMACOAT^®^ 606 solution (case (e)) leads to a ratio of normal to tangential contact force of less than one. The explanation for this is the lower capillary forces acting only in the normal direction, with a simultaneous sharp increase in the viscous forces in the tangential direction. The results for case a) with a reduced liquid loading and case (c) with a reduced surface tension differ only slightly from the second case with 5 vol.-% water.

## 5. Conclusions

In this work, the dynamics of wet particles in a fluidized bed rotor granulator was investigated using CFD-DEM simulation. A liquid bridge model was implemented in DEM to account for the acting physical adhesion mechanisms due to the capillary and viscous forces. In general, the dynamics of the wet particles are affected by the rotation of the plate; therefore, the particles are located near the apparatus wall. The particle concentration is highest in the center of the particle bed and lowest directly above the annular gap. In addition, in the region above the annular gap, due to the inflowing gas, the poloidal velocity of the particles is highest. For the tangential and rotational velocities, the region with high velocities is mainly directly above the rotation plate.

The following findings regarding the influence of liquid loading and liquid properties on the particle dynamics and interactions were obtained:

- Increasing the viscosity to the value of a 6 mass-% PHARMACOAT^®^ 606 coating solution results in a denser particle bed. In addition, the particle rotation velocities and the particle movement in the poloidal plane are reduced.
- A reduced liquid loading in the bed as well as a reduced surface tension of the coating liquid lead to lower capillary forces, and thus, to increased particle movement.
- The fraction of high contact velocities increases at low liquid loading or low surface tension, while it decreases at high viscosity. On the other hand, the average contact force increases significantly with high viscosity.
- Based on the proportional dependence of capillary force on surface tension or viscosity force on viscosity, it was found that an increase in viscosity leads to an increase in aggregate size, whereas a reduction in surface tension results in a decrease.

## Figures and Tables

**Table 1:** Gas properties for the CFD simulation.

Parameters of the Gas	Unit	Value
Fluid	-	air
Fluid temperature	°C	20
Fluid kinematic viscosity	kg∙m^−1^∙s^−1^	$1.58\times 10^{-5}$
Fluid density	kg∙m^−3^	1.2
Inlet gap velocity	m∙s^−1^	32

**Table 2:** Material properties for the DEM simulation.

Parameter	Unit	Value/Variation Range
Particle	-	ceramic cores coated with PVB
Particle bed mass	kg	1.0
Particle density	kg∙m^−3^	3485
Particle diameter	mm	2.8
Young’s modulus particle	GPa	1.69
Young’s modulus walls	GPa	3.0
Poisson ratio	-	0.3
Restitution coefficient	-	0.89
Static friction particle–particle	-	0.23
Static friction particle–wall	-	0.46
Rolling friction particle–particle	-	0.075
Rolling friction particle–wall	-	0.065
Liquid volume on particle	vol-%	1–5
Surface tension	mN∙m^−1^	42.5–72.8
Liquid dynamic viscosity	Pa∙s	0.001–0.619
Liquid density	kg∙m^−3^	1000
Contact angle particle-particle	°	25
Contact angle particle–wall/rotor	°	45

**Table 3:** The five simulation cases with different liquid loading and properties at a fluidization flow of 200 m^3^∙h^−1^, a rotation velocity of the rotor plate of 100 rpm and a bed mass of 1 kg.

Case	Liquid Properties
(a) 1.0 vol.-%	1 vol.-%, γ = 72.8 mN∙m^−1^, η = 1 mPas
(b) 5.0 vol.-%	5 vol.-%, γ = 72.8 mN∙m^−1^, η = 1 mPas
(c) γ = 42.5 mN∙m^−1^	5 vol.-%, γ = 42.5 mN∙m^−1^, η = 1 mPas
(d) η = 61.9 mPa∙s	5 vol.-%, γ = 72.8 mN∙m^−1^, η = 61.9 mPas
(e) γ = 42.5 mN∙m^−1^, η = 61.9 mPa∙s	5 vol.-%, γ = 42.5 mN∙m^−1^, η = 61.9 mPas

**Table 4:** The particle rotation number and the radial movement proportion for the five simulation cases.

Case	PRN in 1/s	RMP in %
(a) 1.0 vol.-%	0.73	6.86
(b) 5.0 vol.-%	0.76	6.01
(c) γ = 42.5 mN∙m^−1^	0.73	7.21
(d) η = 61.9 mPa∙s	0.87	4.58
(e) γ = 42.5 mN∙m^−1^, η = 61.9 mPa∙s	0.87	5.14

**Table 5:** Contact rate and average number of contact partners for the five simulation cases.

Case	Contact Rates/-	Average Numbers of Contact Partners/-
(a) 1.0 vol.-%	2.14	3.23
(b) 5.0 vol.-%	2.96	3.45
(c) γ = 42.5 mN∙m^−1^	2.21	3.34
(d) η = 61.9 mPa∙s	2.56	3.76
(e) γ = 42.5 mN∙m^−1^, η = 61.9 mPa∙s	2.24	3.43

**Table 6:** Average contact velocities and forces for the five simulation cases in contacts between particles (P–P), particles with the cylindrical wall (P–W) and particles with rotor plate (P–R).

Contact Partners	Contact Velocity/m∙s^−1^	Normal Contact Force/mN	Tangential Contact Force/mN
(a) 1.0 vol.-%			
P–P	0.040	1.40	0.25
P–W	0.046	1.52	0.62
P–R	0.208	4.01	1.36
(b) 5.0 vol.-%			
P–P	0.041	1.47	0.26
P–W	0.066	1.58	0.63
P–R	0.217	3.89	1.34
(c) 𝛾 = 42.5 mN∙m−1			
P–P	0.045	1.24	0.22
P–W	0.079	1.18	0.46
P–R	0.230	3.29	1.16
(d) 𝜂 = 61.9 mPa∙s			
P–P	0.033	1.68	0.46
P–W	0.039	1.32	0.77
P–R	0.205	3.59	2.88
(e) 𝛾 = 42.5 mN∙m−1, 𝜂 = 61.9 mPa∙s			
P–P	0.037	1.53	0.43
P–W	0.069	1.12	0.82
P–R	0.222	3.27	3.73

## Data Availability

The data presented in this study are available on request from the corresponding author.
